# Paleotemperature record of the Middle Devonian Kačák Episode

**DOI:** 10.1038/s41598-021-96013-3

**Published:** 2021-08-16

**Authors:** Thomas J. Suttner, Erika Kido, Michael M. Joachimski, Stanislava Vodrážková, Monica Pondrelli, Carlo Corradini, Maria G. Corriga, Luca Simonetto, Michal Kubajko

**Affiliations:** 1 Department of Geological and Environmental Sciences, Appalachian State University, Boone, NC 28608-2006 USA; 2 GeoZentrum Nordbayern, Friedrich-Alexander Universität Erlangen-Nürnberg, Schlossgarten 5, 91054 Erlangen, Germany; 3 Czech Geological Survey, Geologická 6, 152 00 Prague 5, Czech Republic; 4 IRSPS, Università d’Annunzio, viale Pindaro 42, 65127 Pescara, Italy; 5 Dipartimento di Matematica e Geoscienze, Università degli Studi di Trieste, Via Weiss 2, 34128 Trieste, Italy; 6 Museo Friulano di Storia Naturale, via Sabbadini 22-32, 33100 Udine, Italy

**Keywords:** Biogeochemistry, Climate change, Palaeoclimate

## Abstract

The Middle Devonian Epoch, ~ 393–383 million years ago, is known for a peak in diversity and highest latitudinal distribution of coral and stromatoporoid reefs. About 388 million years ago, during the late Eifelian and earliest Givetian, climax conditions were interrupted by the polyphased Kačák Episode, a short-lived period of marine dys-/anoxia associated with climate warming that lasted less than 500 kyr. Reconstruction of the seawater temperature contributes to a better understanding of the climate conditions marine biota were exposed to during the event interval. To date, conodont apatite-based paleotemperatures across the Eifelian–Givetian boundary interval have been published from Belarus, France, Germany and North America (10–36° S paleolatitude). Here we provide new δ^18^O_apatite_ data from the Carnic Alps (Austria, Italy) and the Prague Synform (Czech Republic). For better approximation of the paleotemperature record across the Kačák Episode, a latitude-dependent correction for Middle Devonian seawater δ^18^O is applied. Because δ^18^O_apatite_ data from shallow marine sections are influenced by regional salinity variations, calculated mean sea surface temperatures (SST) are restricted to more open marine settings (22–34° S paleolatitude). Water temperatures reach ~ 34 °C in the Prague Synform and ~ 33 °C in the Carnic Alps and suggest that SSTs of the southern hemisphere low latitudes were ~ 6 °C higher than previously assumed for this time interval.

## Introduction

The Middle Devonian Kačák Episode^1^ is known as a minor global extinction event associated with a eustatic sea level rise^2^. The event interval, first recognized as significant lithological change from the Choteč Limestone to the Kačák Shale within the Prague Synform (Czech Republic), is characterized by global occurrence of bottom-water dys-/anoxia and expanded deposition of black shales^1,3–9^. The Kačák Episode was divided into two event intervals, named late Eifelian 1 Event (LEE1) and late Eifelian 2 Event (LEE2), reflecting its polyphased nature^1,10^. Although the Kačák Episode was initiated by black shale deposition during the late Eifelian about 388 million years ago (uppermost part of the *kockelianus* conodont Biozone), it did not terminate at the Eifelian–Givetian boundary but reached into the earliest Givetian (*hemiansatus* conodont Biozone)^10,11^. At the Eifelian/Givetian GSSP (Global Stratotype Section and Point) at Mech Irdane (Morocco), the Kačák Episode is temporally constrained to a relatively short interval of about 370 kyr^12,13^.

Biodiversity analysis has shown that mainly ammonoids^5,14–16^, conodonts^10,11,17,18^ and trilobites^19,20^ suffered from a certain taxonomic loss. However, it appears that extinction was not the main response of faunal communities to environmental changes but rather habitat tracking and speciation events. Several groups even produced new taxa, e.g., planktonic dacryoconarid tentaculitids like *Nowakia otomari* occurred with the onset of LEE1^21^. An increased intraspecific variability is observed among some polygnathid and icriodontid conodont taxa^10,11,18,22^. Additionally, the bathymetric changes coherent with the late Eifelian eustatic sea level rise effected the paleogeographic distribution of benthic marine organisms^23,24^. Some of Rhenish-type brachiopod and trilobite taxa migrated from southern Euramerica (Ardenne area) to the northern shelf of Gondwana (Prague Synform) during the latest Eifelian^24^. Similar migration patterns are seen among rugose corals along the western Euramerican shallow shelf, where cosmopolitan taxa of the Old World Realm invaded and replaced the endemic coral community of the East Americas Realm^23^. Probably, not only because previously existing topographic barriers were removed and shelf areals broadened, but also because the inferred global warming allowed/forced extension of the latitudinal distribution of temperature-dependent taxa.

New insights about the late Eifelian global warming period known as the Kačák Episode were generated by analysis of latitudinally dependent paleotemperature patterns reconstructed from conodont apatite. So far, paleotemperatures were published from southern hemisphere low latitudes between 10° and 36° S for localities in France (Pic de Bissous)^25^ and Germany (Blankenheim, Hillesheimer Mulde, Schönecken-Dingdorf and Blauer Bruch)^25,26^. Additional data from Nevada, USA (northern Antelope mountains) documented a negative shift in the δ^18^O_apatite_ during the onset of the LEE1 (upper part of the *kockelianus* Biozone)^27^, and more recently, paleotemperature reconstructions from Belarus were provided for the latest Eifelian interval (*ensensis* Biozone)^28,29^.

**Figure 1:**
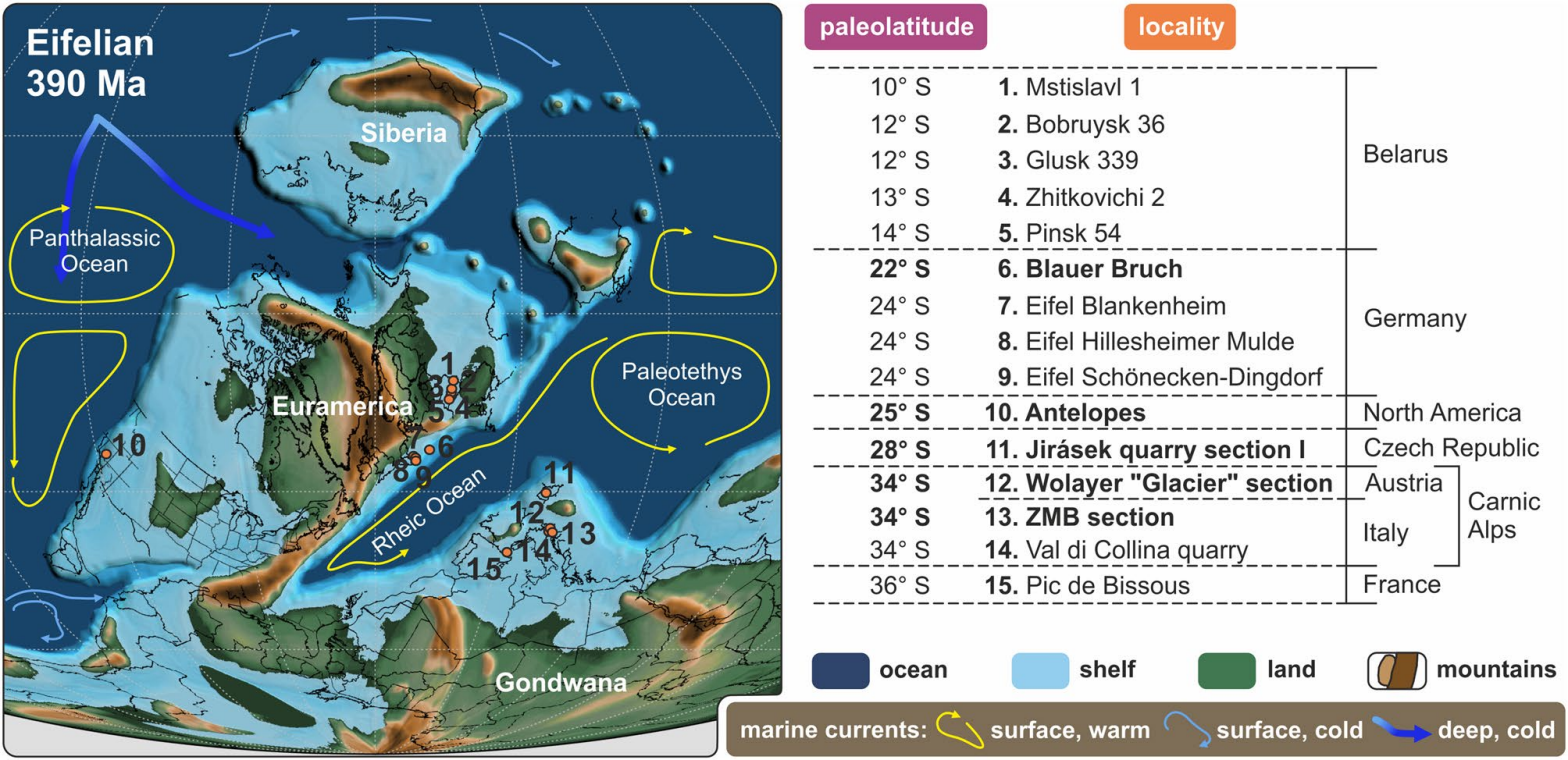
Paleolatitudinal position of published and newly studied localities during the Middle Devonian (late Eifelian to earliest Givetian). Localities reflecting more open marine conditions are marked in bold. Eifelian paleogeographic reconstruction modified after Scotese^33^.

Here we discuss late Eifelian to earliest Givetian paleotemperature estimates based on new δ^18^O_apatite_ data from a shallow to deeper water transect within the Carnic Alps (Austria, Italy) and an offshore section from the Prague Synform (Czech Republic). Together with palaeotemperature estimates from other localities within the Rheic Ocean and the North American shelf, mean sea surface temperatures for each region and biozone are calculated and compared (Fig. 1). Because paleotemperature estimates from shallow marine localities appear to be biased by varying salinities, the temperature record across the Kačák Episode is limited to more open marine deposits of the Carnic Alps.

Keeping in mind that Middle Devonian coral-stromatoporoid reefs showed a remarkable wide latitudinal dispersal of up to 50° on either hemisphere with an acme in diversity just after the Kačák Episode during the early and middle Givetian (~ 387–382 million years ago)^30–32^, only a detailed study of the event interval can show how the marine biosphere dealt with environmental changes at that time. Estimation of paleotemperatures across the event interval contributes to uncover critical temperatures and to identify ecological limits of climate sensitive low latitude communities.

## Geological settings

**Figure 2:**
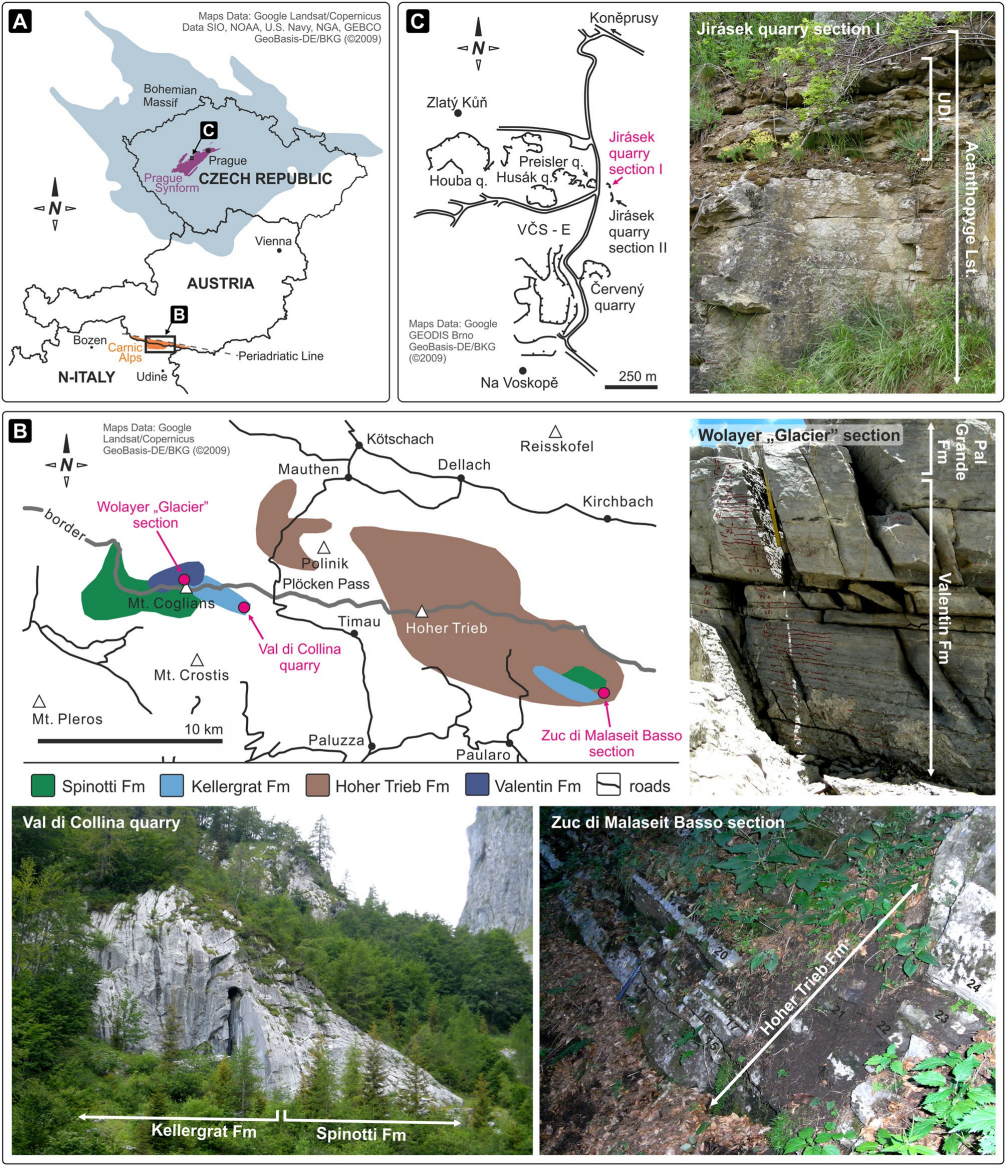
(**A**) Location of the Carnic Alps at the Austro-Italian border and the Prague Synform in Czech Republic. (**B**) Simplified map of the central Carnic Alps with areas of occurrence of the formations analysed in this paper. Investigated sections are the Wolayer “Glacier” section (Valentin Formation), the Val di Collina quarry (Spinotti and Kellergrat formations) and the Zuc di Malaseit Basso section (Hoher Trieb Formation). (**C**) Detailed map of the Koněprusy area with location of the Jirásek quarry section I and II. The photo left of the map shows the Upper Dark Interval (UDI) of the uppermost part of the Acanthopyge Limestone at Jirásek quarry section I. Maps in (**A**–**C**) designed in CorelDRAW^®^ X8 based on google earth images (maps data attribution incorporated within illustrations).

The Carnic Alps, part of the Proto-Alps^34^, were situated on the northern peri-Gondwana shelf located around 34° S during the Devonian. Today the area forms a more than 2000 m high mountain chain along the Austro-Italian border with Middle Devonian rocks well-exposed between the Biegengebirge in the west and Mount Osternig in the east (Fig. 2A,B)^35^. Coeval sections from different bathymetric settings were investigated (Fig. 2B). The section studied in the Val di Collina quarry (N46° 35′ 49.07″; E12° 55′ 29.22″) consists of back reef and reef environments belonging to the Spinotti and Kellergrat formations, respectively. The Zuc di Malaseit Basso (ZMB) section (N46° 33′ 19.06″; E13° 11′ 10.6″) was deposited within a distal slope setting of the Hoher Trieb Formation, while the Wolayer “Glacier” section (N46° 36′ 46.56″; E12° 52′ 33.66″) is represented by condensed pelagic deposits of the Valentin Formation. These sections were correlated using high-resolution conodont biostratigraphy^11^.

The Middle Devonian Srbsko Formation in the Prague Synform (part of the Bohemian Massif) is generally developed in siliciclastic facies^36^ and was located around 28° S. The studied Jirásek quarry section I (N49° 54′ 50.2″; E14° 04′ 34.2″) represents a unique section where the stratigraphic equivalent of the Kačák Member of the Srbsko Formation is developed in carbonate facies. The so-called Upper Dark Interval (UDI), exposes a dark gray thin-bedded to nodular limestone interval representing the uppermost portion of the Acanthopyge Limestone (Choteč Formation, Fig. 2A,C)^37^. Upper Eifelian deposits are unconformably overlain by a limestone breccia, wherefore the middle/upper? part of the *ensensis* Biozone within the UDI was probably reworked^18^.

## Results

**Figure 3:**
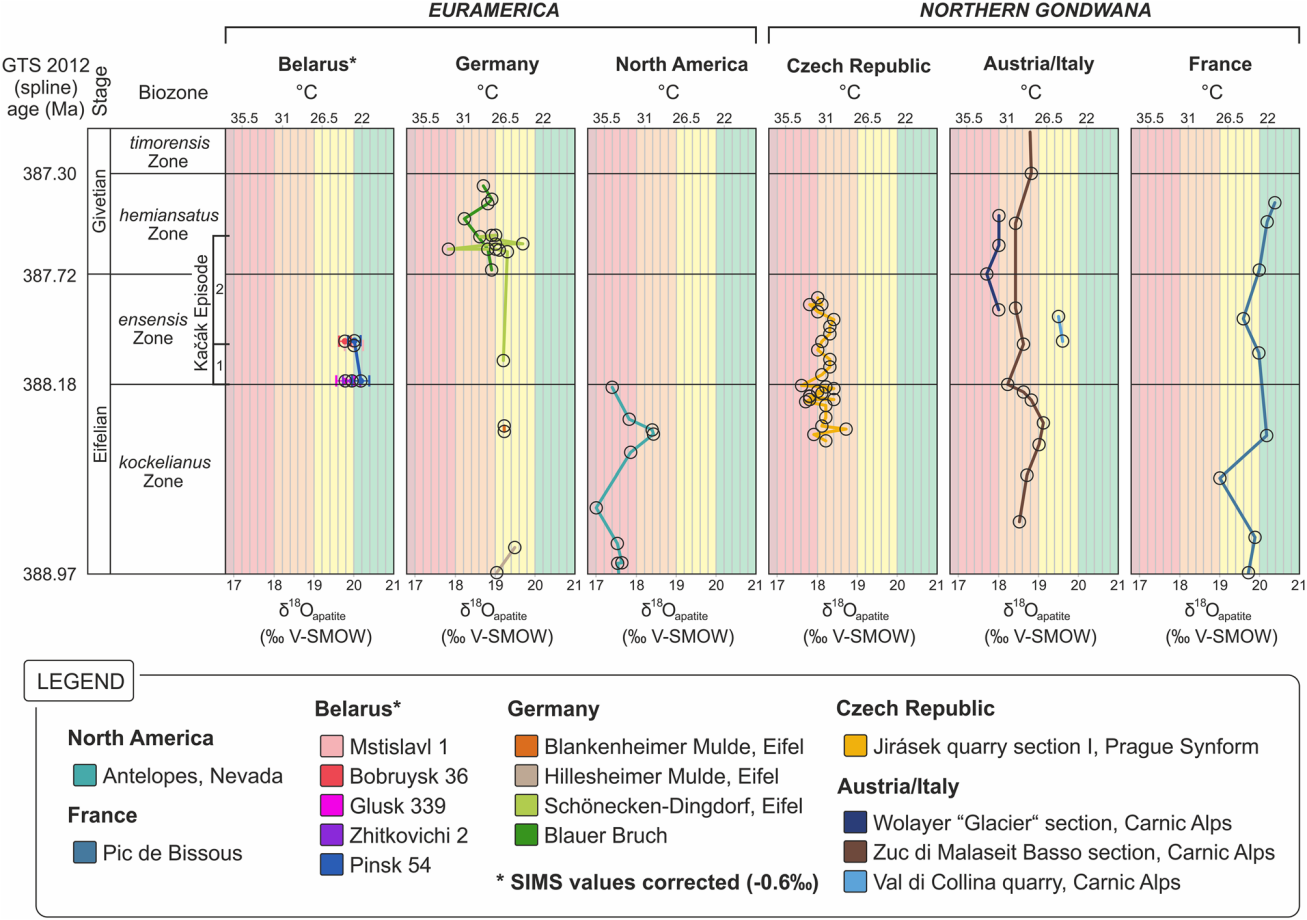
Comparison of δ^18^O_apatite_ isotope records during the late Eifelian and earliest Givetian (North–South sorted). Data from Belarus, Germany, North America and France compiled from Elrick et al.^27^, Joachimski et al.^25^, Königshof et al.^26^ and Narkiewicz et al.^28^.

In the Carnic Alps, the δ^18^O_apatite_ record of the ZMB section includes 11 measurements covering an interval between the lower part of the *kockelianus* Biozone (late Eifelian) and the entry of the *timorensis* Biozone (early Givetian). δ^18^O_apatite_ values are between 18.2 to 19.1‰ (VSMOW). Oxygen isotopes show a weak positive shift of 0.6‰ in the *kockelianus* Biozone, followed by a negative shift of 0.9‰ in the upper part of this biozone and gradually increasing to values of 18.8‰ at the *hemiansatus*/*timorensis* Biozone boundary (Fig. 3; Supplementary Fig. 1). Only few δ^18^O_apatite_ data were generated from shallow marine deposits due to the generally lower abundance of conodont elements. Samples from the Val di Collina quarry record only little variation during a short interval within the *ensensis* Biozone with values of 19.5 and 19.6‰ (Fig. 3; Supplementary Fig. 1). The condensed pelagic sequence of the Wolayer “Glacier” section yielded abundant conodonts, which were extracted from cm-thick limestone levels separated along slightly uneven stylolite layers. δ^18^O_apatite_ values measured across the Eifelian–Givetian boundary vary between 17.7 and 18.0‰ (Fig. 3; Supplementary Fig. 1).

In the Prague Synform, the δ^18^O_apatite_ record at the Jirásek quarry section I includes 28 measurements covering the upper part of the *kockelianus* Biozone and most of the *ensensis* Biozone. Values range between 17.6 to 18.7‰ (VSMOW). The highest value of 18.7‰ is observed during the late *kockelianus* Biozone while the lowest value is observed before the entry of the *ensensis* Biozone (Fig. 3; Supplementary Fig. 1). Variations of about 1‰ are recognized within the sampled interval.

## Discussion

For a better understanding of the paleotemperature record across the E/G boundary, results from this study are compared with published δ^18^O_apatite_ values^25–28^ (Fig. 3). In total, 67 measurements are documented from the late Eifelian (*kockelianus* and *ensensis* biozones) and 21 values from the earliest Givetian (*hemiansatus* Biozone). The highest resolution is documented for the late Eifelian with 28 δ^18^O_apatite_ values reported from the Prague Synform (28° S). All data included in this study derive from localities allocated in southern hemisphere latitudes between ~ 10–36° S. In addition to the common approach used for paleotemperature calculation from δ^18^O_apatite_, we applied a latitude-dependent correction for δ^18^O_seawater_ following the isotope-enabled ocean–atmosphere general circulation model of the National Aeronautics and Space Administration Goddard Institute for Space Studies (NASA GISS) ModelE-R^38^. The model provides better constraints on variations in δ^18^O_seawater_ as consequence of latitude-dependent changes in the evaporation vs. precipitation ratio. A higher evaporation vs. precipitation ratio in the subtropics results in higher salinities and thus a higher δ^18^O_seawater_ whereas equatorial and higher latitudes are characterized by a lower δ^18^O_seawater_. We applied the correction published for the early Paleocene, a relatively warm time interval, likely comparable to the Devonian.

### Paleotemperature estimates

For paleotemperature calculation, the phosphate-water isotope fractionation equation of Lécuyer et al.^39^ is used. Because no or at least no permanent glacial ice sheet was established around 388 million years ago^40,41^ and flooded continental area reached a maximum within the entire Phanerozoic record during the Middle Devonian^42^, a δ^18^O value of − 1‰ VSMOW for Middle Devonian seawater is assumed. As result, δ^18^O_apatite_ data from eastern and southern Euramerica indicate lower temperatures compared with localities from more southern latitudes on the western shelf of Euramerica and northern Gondwana. Depending on data availability, calculated mean δ^18^O_apatite_ values of each area from north to south show highest temperatures of ~ 33 °C on the western shelf of Euramerica around 25° S during the pre-/onset of the LEE1 (*kockelianus* Biozone), ~ 32 °C on the northern Gondwana shelf at 28° S during the LEE1-LEE2 interval (*ensensis* Biozone) and ~ 31 °C within offshore deposits at 34° S during the late/post-LEE2 (*hemiansatus* Biozone) (Table 1). Mean paleotemperatures during the pre-/onset of the LEE1 are ~ 7 °C higher in western Euramerica at 25° S than in southern Euramerica at 24° S. The mean temperature difference between localities on the southern shelf of Euramerica (24° S) and northern Gondwana (28° S) during the LEE1–LEE2 interval is 5 °C. In the earliest Givetian (late/post-LEE2), the temperature offset between 24° S and 34° S decreases to ~ 4 °C. However, a gradual latitudinal decrease in mean sea surface temperature is observed between 25 and 34° S during the late Eifelian and earliest Givetian Kačák Episode (Table 1).

**Table 1:** Calculated regional paleotemperature﻿ ranges and mean values for each paleolatitude/country and biozone.

Paleo-latitude	Country	Paleotemperature estimates (δ^18^O_seawater_ = − 1‰ VSMOW)								
		Pre-/onset LEE1			LEE1–LEE2			Late/post-LEE2		
		Min–max	Mean	n	Min–max	Mean	n	Min–max	Mean	n
10–14° S	Belarus	–	–	–	22–24 °C	23 °C	6	–	–	–
22° S	Germany^a^	–	–	–	–	–	–	28–31 °C	29 °C	6
24° S	Germany^b^	25–27 °C	27 °C	4	27 °C	27 °C	1	24–33 °C	28 °C	9
25° S	USA	30–36 °C	33 °C	9	–	–	–	–	–	–
28° S	Czech Republic	29–34 °C	32 °C	16	30–33 °C	32 °C	12	–	–	–
34° S	Austria	–	–	–	32–33 °C	32 °C	2	32 °C	32 °C	2
34° S	Italy^a^	27–31 °C	29 °C	7	29–30 °C	30 °C	2	30 °C	30 °C	1
34° S	Italy^b^	–	–	–	25 °C	25 °C	2	–	–	–
36° S	France	22–27 °C	24 °C	4	23–25 °C	24 °C	2	21–23 °C	22 °C	3
Min–max		22–36 °C			22–33 °C			21–33 °C		
Mean		27–31 °C	30 °C	40	27–28 °C	28 °C	27	27–30 °C	28 °C	21
Min–max*		27–36 °C			29–33 °C			30–32 °C		
Mean*		28–34 °C	31 °C	32	27–32 °C	31 °C	16	29–31 °C	30 °C	9

Pre-/onset LEE1 = *kockelianus* Biozone; LEE1–LEE2 = *ensensis* Biozone; late/post-LEE2 = *hemiansatus* Biozone; Germany: a = Blauer Bruch, b = Eifel; Italy: a = ZMB section, b = Val di Collina quarry; min–max* and mean* indicate calculation excluding shallow marine localities: Belarus, Germany^b^, Italy^b^ and France. Remark: Overall mean SST of the Carnic Alps (summarizing localities in Austria and Italy^a^) are 31 °C for the *ensensis* and *hemiansatus* biozones.

### Latitude correction for δ^18^O_seawater_

**Figure 4:**
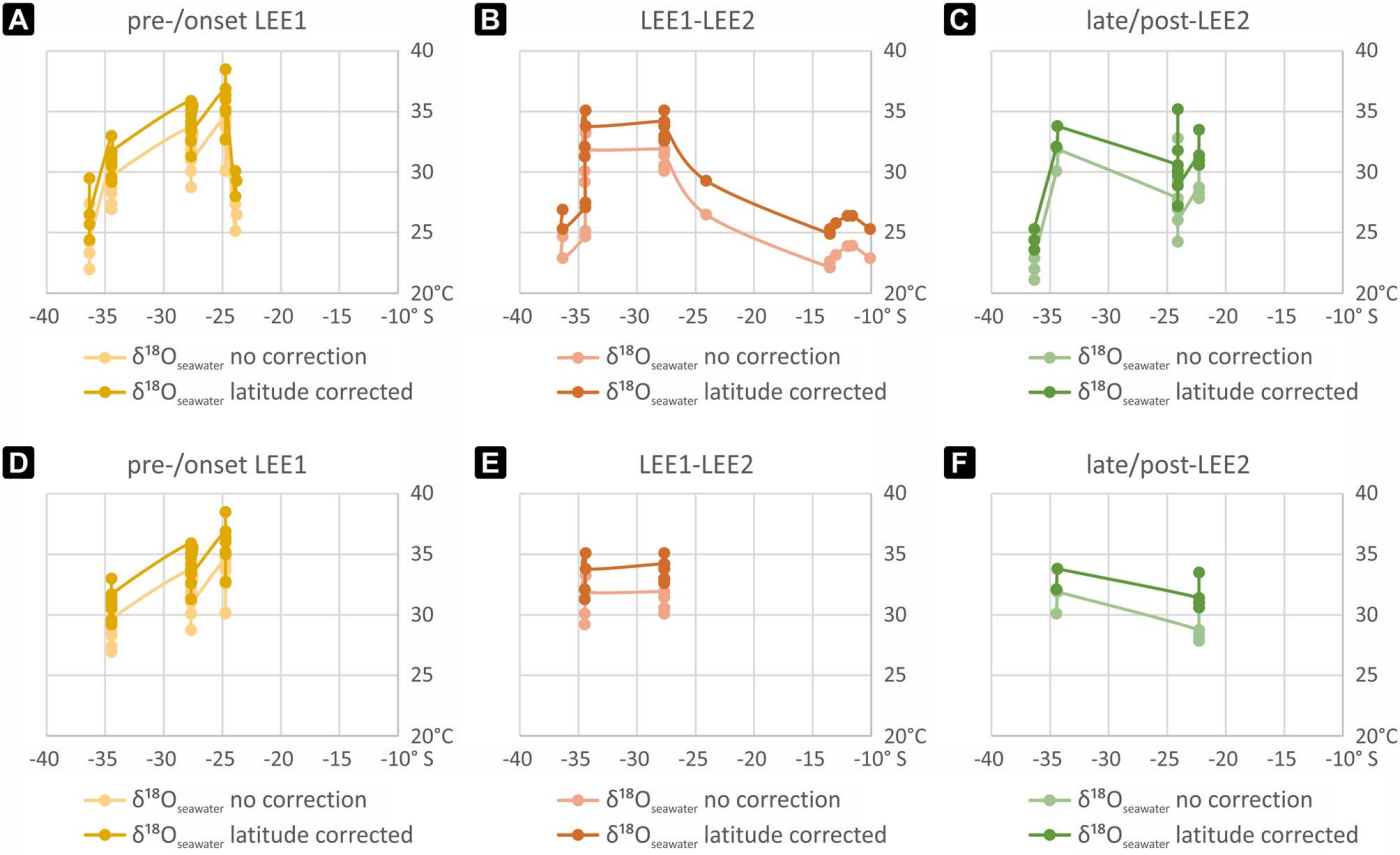
Comparison of paleotemperatures calculated from δ^18^O_apatite_ assuming δ^18^O_seawater_ = − 1‰ VSMOW vs. applying a latitude correction for δ^18^O_seawater_ during the (**A**,**D**) pre-/onset LEE1 (*kockelianus* Biozone), (**B**,**E**) LEE1–LEE2 (*ensensis* Biozone) and (**C**,**F**) late/post-LEE2 (*hemiansatus* Biozone). (**A**–**C**) Entire dataset. (**D**–**F**) Shallow marine localities excluded. x-axis: southern hemisphere latitude; y-axis: temperature in °C.

Since the δ^18^O of seawater varies in part due to the evaporation to precipitation ratio, we calculated latitude dependent δ^18^O_seawater_ values using the GISS ModelE-R provided by Roberts et al.^38^ (Table 2). A comparison of both approaches (Tables 1 vs. 2) shows ~ 2 °C higher temperatures when applying a latitude correction for δ^18^O_seawater_ (Fig. 4A–C; Supplementary Fig. 2). Hottest mean temperatures of ~ 36 °C at 25° S during the pre-/onset of the LEE1 (*kockelianus* Biozone), ~ 34 °C at 28° S during the LEE1-LEE2 interval (*ensensis* Biozone) and ~ 33 °C at 34° S during the late/post-LEE2 (*hemiansatus* Biozone) were calculated using the data available for each biozone. Since SSTs from epeiric seas and continental margins are generally higher than average temperatures in the same latitude band^43^, we conclude that mean SSTs did not reach beyond 36 °C within southern hemisphere low latitudes during the polyphased Kačák Episode (Table 2).

**Table 2:** Paleolatitude corrected regional paleotemperature including the latitude correction values for δ^18^O_seawater_ and mean values calculated for each paleolatitude/country and biozone.

Paleo-latitude	Country	Lat. corr. δ^18^O_seawater_	Paleolatitude corrected paleotemperature estimates								
			Pre-/onset LEE1			LEE1–LEE2			Late/post-LEE2		
			Min–max	Mean	n	Min–max	Mean	n	Min–max	Mean	n
10–14° S	Belarus	− 0.70 to − 0.63	–	–	–	25–26 °C	26 °C	6	–	–	–
22° S	Germany^a^	− 0.53	–	–	–	–	–	–	31–34 °C	31 °C	6
24° S	Germany^b^	− 0.54	28–30 °C	29 °C	4	29 °C	29 °C	1	27–35 °C	30 °C	9
25° S	USA	− 0.54	33–39 °C	36 °C	9	–	–	–	–	–	–
28° S	Czech Republic	− 0.56	31–36 °C	34 °C	16	33–35 °C	34 °C	12	–	–	–
34° S	Austria	− 0.66	–	–	–	34–35 °C	35 °C	2	34 °C	34 °C	2
34° S	Italy^a^	− 0.66	29–33 °C	31 °C	7	31–32 °C	32 °C	2	32 °C	32 °C	1
34° S	Italy^b^	− 0.66	–	–	–	27–28 °C	27 °C	2	–	–	–
36° S	France	− 0.70	24–30 °C	27 °C	4	25–27 °C	26 °C	2	24–25 °C	24 °C	3
Min–max			24–39 °C			25–35 °C			24–35 °C		
Mean			29–33 °C	33 °C	40	29–30 °C	31 °C	27	30–32 °C	30 °C	21
Min–max*			29–39 °C			31–35 °C			32–34 °C		
Mean*			31–36 °C	34 °C	32	33–34 °C	34 °C	16	33 °C	32 °C	9

Pre-/onset LEE1 = *kockelianus* Biozone; LEE1–LEE2 = *ensensis* Biozone; late/post-LEE2 = *hemiansatus* Biozone; Germany: a = Blauer Bruch, b = Eifel; Italy: a = ZMB section, b = Val di Collina quarry; min–max* and mean* calculated excluding shallow marine localities: Belarus, Germany^b^, Italy^b^ and France. Lat. corr. = latitude correction. Overall mean SST of the Carnic Alps (summarizing localities in Austria and Italy^a^) are 33 °C for the *ensensis* and *hemiansatus* biozones.

### Uncertainties in the latitudinal paleotemperature record

#### High SSTs of Devonian low latitudes

A recently published Devonian climate simulation suggests a global annual mean surface air temperature of 21 °C and tropical SST of 28 °C for the Middle Devonian^44^. The model^44^ suggests colder low latitude mean SSTs compared to SSTs calculated from δ^18^O_apatite_ of Devonian conodonts. Combining the new δ^18^O_apatite_ data from the Prague Synform (28° S) and the Carnic Alps (34° S) with other published data (Supplementary Fig. 2), mean tropical-subtropical SSTs reach 33 °C during late Eifelian/earliest Givetian time (Table 2: mean). However, Judd et al.^43^ call for caution when using paleotemperatures from shallow epeiric seas and continental margins as these are systematically higher compared to open-ocean temperatures from the same latitude band and thus result in an overestimation of global mean temperatures. Though, it seems a paradox, that especially conodonts from epeiric Belarus (10–14° S), the Eifel area (24° S), shallow marine platform deposits of the Carnic Alps (34° S) and from Pic de Bissous (36° S) indicate significantly lower paleotemperatures compared to those from slope and offshore deposits. A possible explanation could be an interval of arid climate conditions in tropical-subtropical realms linked with the Kačák Episode which led to increased evaporation in shallow shelf areas and thus higher δ^18^O_apatite_ values due to regionally increased salinity.

#### Zonal heterogeneity of low latitude SSTs

δ^18^O_apatite_ from the epeiric sea of eastern Euramerica (10–14° S) and shallow marine deposits of southern Euramerica (24° S) exhibit higher values which compare better with values of conodonts from northern Gondwana at 34–36° S rather than with those from more open marine settings between 25 and 34° S at least during the late Eifelian (Fig. 3, Supplementary Fig. 2). This could be explained by regional salinity variations as suggested for localities between 10 and 14° S^28,29^ or upwelling of colder intermediate waters in the Rheic Ocean^45–48^.

SSTs modelled for the Middle Devonian^44^ give significantly lower SSTs along the southern hemisphere Euramerican shelf compared to temperature estimates for open ocean settings of the same latitude. According to the Holocene SST record, a highest temperature of 32 °C (central equatorial Indian Ocean) and a lowest temperature of 17 °C (eastern equatorial Atlantic) between 0 and 5° S are documented^49^. As indicated by modern sea surface temperatures^50^, SST of low latitude waters does not always show a gradational temperature decrease towards higher latitudes. Hence, temperature variations within the low latitude belt like those documented in δ^18^O_apatite_ from Euramerican shallow shelf localities between 10 and 24° S likely reflect, similar to present day SSTs, regional factors as for example evaporation, runoff via large river delta systems, submarine groundwater discharge, karst water flow in coastal areas, ocean circulation, or upwelling zones along continental shelfs.

#### Mean SST record across the Eifelian/Givetian boundary

The interpretation of the paleotemperature trend across the Eifelian/Givetian boundary is difficult, because of the low number of isotope values available. Data of only two localities, at 34° S (ZMB section, Italy) and 36° S (Pic de Bissous sections, France), span the entire interval from the *kockelianus* until the *hemiansatus* Biozone. Additionally, δ^18^O_apatite_ values from the epeiric Belarusian Basin (10–14° S)^28,29^, as well as those derived from conodonts of shallow marine deposits at 24° S^25,26^, 34°S (this study, Supplementary Fig. 2) and 36° S^25^ are higher compared to values from conodonts of coeval more open marine habitats, contradicting expected values in relation to the latitudinal allocation. δ^18^O_apatite_ values of the three localities from different depositional settings within the Carnic Alps at 34° S, for example, differ significantly. Local conditions could have been responsible for such high zonal heterogeneity of SST. While the shallow marine carbonate platform setting (Val di Collina section) might have been affected by higher evaporation translating into higher δ^18^O_seawater_ and thus higher δ^18^O_apatite_ values, conodonts from pelagic deposits (Wolayer “Glacier” section) show lowest δ^18^O_apatite_ values likely reflecting normal-marine more open ocean salinity and temperature. This documents that δ^18^O_apatite_ values obtained from conodonts of tropical-subtropical shallow marine settings may not only represent seawater temperature but as well local salinity variations, as noted by several studies on δ^18^O_apatite_ before^25,27–29,43,51,52^. Thus, exclusively δ^18^O_apatite_ data from more open marine settings are used for calculation of mean paleotemperature. This results in higher mean SST as observed especially for mean SST calculated for the LEE1-LEE2 interval (*ensensis* Biozone), with a significant increase in temperatures from ~ 31 °C to ~ 34 °C (Table 2: mean vs. mean*). In summary, our data argue for at least 6 °C higher mean SST across the Kačák Episode compared to published model estimates suggesting mean values of 28 °C for the tropical-subtropical climate belt^44^.

Additionally, more open marine sections at 25–34° S show slightly decreasing mean SST from north to south during each biozone-interval (Fig. 4A–C vs. D–F, Table 2). A mean latitudinal temperature gradient of ~ 5 °C between 25 and 34° S is observed for the pre-/onset LEE1 interval (*kockelianus* Biozone) which is somewhat higher compared to the present day mean temperature gradient of ~ 4.5 °C for the same latitudinal range^43^. Calculating a mean latitudinal temperature gradient between 28 and 34° S, a decrease from ~ 3 °C during the pre-/onset LEE1 (*kockelianus* Biozone) to ~ 1 °C during the LEE1–LEE2 (*ensensis* Biozone) is observed (compare Fig. 4D–E). The present-day mean latitudinal temperature gradient between 28 and 34° S is ~ 3.3 °C^43^. Thus, we argue that global warming resulted in a minor latitudinal SST gradient in the subtropics and probably slightly higher latitudes that may have had an important effect for marine biota. Specifically, climate sensitive organisms like corals and stromatoporoids could spread more easily towards higher latitudes along broad shelfs. Hence, we suggest that the Kačák Episode could have triggered the early-middle Givetian acme of coral distribution and diversity.

## Conclusion

The main results of this study can be summarized as follows:

- New conodont δ^18^O_apatite_ data around the Kačák Episode (late Eifelian to earliest Givetian) from the Carnic Alps (34° S) and the Prague Synform (28° S) indicate mean SSTs between 31 and 34 °C.
- SST of late Eifelian southern hemisphere low latitudes was at least 6 °C higher than previously assumed.
- δ^18^O_apatite_ based paleotemperatures from conodonts of tropical-subtropical shallow water should only be used with great caution, confirming previous studies suggesting that salinity variations may affect the oxygen isotope values.
- Global warming caused a flattening of the latitudinal temperature gradient in the subtropics and probably as well higher latitudes having an important effect for marine biota.
- Specifically, climate sensitive organisms could spread more easily towards higher latitudes along broad shelfs. Hence, we suggest that the Kačák Episode could have triggered the early-middle Givetian acme of coral and stromatoporoid distribution and diversity.

## Materials and methods

### Conodont extraction

Conodont elements were extracted from limestones using 5% formic acid (Carnic Alps 2013, 2015; Prague Synform 2013) and 6% acetic acid (Prague Synform 2020) diluted in 5 L buckets filled with tap water. In order to prevent dissolution of conodont elements, the acid was exchanged twice a day until the carbonates were completely dissolved (generally after 7–10 days). In case of large amounts of insoluble residues, heavy liquid separation was applied at room temperature (sodium polytungstate; density: 2.79 g/cm^3^). The colour alteration index (CAI) of the conodont elements from the Carnic Alps is 4–5 and 5 (Supplementary Fig. 3) which agrees well with earlier observations of Brime et al.^53^, who published a comprehensive conodont CAI study of the Carnic Alps. Conodonts from the Jirásek quarry (Prague Synform) have a CAI of 3 (Supplementary Fig. 3).

### Repository

Conodont elements from the Carnic Alps are stored at the Geological Survey of Austria in Vienna, Austria (Wolayer “Glacier” section: GBA-2016/013/0001-0018; Val di Collina quarry: GBA-2016/015/0001-0015) and at the Museo Friulano di Storia Naturale in Udine, Italy (ZMB section: MFSNgp 48161-48222). Conodont elements from the Prague Synform are stored at the Czech Geological Survey (Jirásek quarry section I and II: SV1-SV121). Permission was obtained from repository holding Institutions for the use of conodont materials included within this study.

### Oxygen isotope analyses

Two analytical methodologies, thermal conversion-elemental analyser-isotope ratio mass spectrometry (TC-EA IRMS) and secondary ionization mass spectrometry (SIMS) are commonly used for measuring oxygen isotopes on biogenic apatite. While the first technique measures the oxygen isotopic composition of the PO_4_ group of several conodont specimens^25^, SIMS enables a high-resolution spot analysis, but measures δ^18^O of total oxygen (PO_4_^3−^, CO_3_^2−^ and OH^−^) of single conodont elements^54^. Oxygen isotopes of bioapatite (conodont crown tissue without basal tissue preserved) within this study were analysed by TC-EA IRMS using a ThermoFinnigan Delta V Plus mass spectrometer at the Geozentrum Nordbayern (FAU Erlangen-Nürnberg, Germany). Depending on the amount of trisilverphosphate precipitated after dissolving conodont apatite in HNO_3_, triplicate measurements were conducted whenever possible. Values are reported in ‰ relative to VSMOW. The standard deviation of replicate sample analyses was ± 0.02 to ± 0.34 (1σ; see Supplementary Fig. 1). NBS 120c was measured as 21.7‰. Literature based SIMS values and TC-EA IRMS values that are calibrated with standards other than NBS 120c (21.7‰) were corrected for direct comparison (see Supplementary Fig. 2: − 0.7‰^25^; − 0.9‰^27^; − 0.6‰^28^). For paleotemperature calculation, the phosphate-water isotope fractionation equation of Lécuyer et al.^39^ is used: T(°C) = 117.4(± 9.5) − 4.50(± 0.43) (δ^18^O_apatite_ − δ^18^O_seawater_) assuming δ^18^O of Devonian seawater as − 1‰ VSMOW.

### Stratigraphic correlation of δ^18^O_apatite_ records

Biostratigraphically constrained δ^18^O_apatite_ values from the Carnic Alps^11^, the Prague Synform^18^ and the Blankenheim section of the Eifel area^26^ are correlated within biozonal boundary ranges via high-resolution magnetic susceptibility and geochemical data. Published data of other sections from Germany and from France^25^ are plotted according to the absolute age^25^ and correlated via conodont biozone boundaries^55–58^. Data from North America^27^ are correlated with the ZMB section of the Carnic Alps^11^ via the *australis*/*kockelianus* biozone boundary and the upper limit of the T-R cycle Id (calibrated to 393.37 Ma)^11^. Data published from Belarus^28^ are biostratigraphically constrained by conodonts to the *ensensis* Biozone and were already combined within the standard-corrected data of Joachimski et al.^25^.

### Calculation of latitude dependent δ^18^O of seawater

Seawater δ^18^O is dependent on the ratio evaporation vs. precipitation which is higher in subtropical latitudes relative to equatorial and higher latitudes. The calculation of spatial gradients in δ^18^O_seawater_ follows Roberts et al.^38^, which prerequisites exact paleolatitude calculation for each locality. Since the climate conception of an ice-free Middle Devonian^40,42^ compares better with early Paleogene climate conditions rather than with preindustrial climates, we adopted latitude-dependent seawater δ^18^O estimates for the early Paleogene^38^.

### Paleolatitude calculation

The calculation of paleolatitudinal positions for the various areas follows Scotese and Wright^59^ (Supplementary Fig. 2). Evaluation of the tectonic setting around Val di Collina quarry^53^ results in a slightly more southern paleolatitudinal position than suggested by Scotese and Wright^59^. In general, the paleo-position is calculated to a latitudinal precision of 1°, based on the equation used.

## Supplementary Information

Supplementary Figures.

## Data Availability

All data of this study are provided in the paper and Supplementary Information.
