# Supplementary material for: Paleotemperature record of the Middle Devonian Kačák Episode
Source: Sci Rep. 2021 Aug 16;11:16559. doi: 10.1038/s41598-021-96013-3 (PMC8368033; doi:10.1038/s41598-021-96013-3)

Supplementary Materials

Supplementary Fig. 1. Raw data: Conodont crown tissue for $\delta^{18}O_{apatite}$ analysis.

sample ID	d18O permil (VSMOW)	std. dev.	n	conodont genera	amount of specimens	Date and Place
Jirásek quarry section I						
30 JI	18,2	0,18	3	*Polygnathus*	100-150 fragmented elements	January 2020, Erlangen
60 M	17,9	0,05	3	*Polygnathus*	100-150 fragmented elements	January 2020, Erlangen
85 JI	18,7	0,09	3	*Polygnathus*	100-150 fragmented elements	January 2020, Erlangen
100 M	18,1	0,13	3	*Polygnathus*	100-150 fragmented elements	January 2020, Erlangen
140 M	18,2	0,22	3	*Polygnathus*	100-150 fragmented elements	January 2020, Erlangen
200 M	18,2	0,20	3	*Polygnathus*	100-150 fragmented elements	January 2020, Erlangen
JI/03	17,7	0,24	3	*Polygnathus*	100-150 fragmented elements	June 2013, Erlangen
JI/04	17,8	0,12	2	*Polygnathus*	100-150 fragmented elements	June 2013, Erlangen
225 JI	18,4	0,11	3	*Polygnathus*	100-150 fragmented elements	January 2020, Erlangen
240 JI	17,8	0,34	3	*Polygnathus*	100-150 fragmented elements	January 2020, Erlangen
JI/05	17,8	0,06	3	*Polygnathus*	100-150 fragmented elements	June 2013, Erlangen
JI/06	18,1	0,23	3	*Polygnathus*	100-150 fragmented elements	June 2013, Erlangen
260 M	18,0	0,09	3	*Polygnathus*	100-150 fragmented elements	January 2020, Erlangen
JI/07	18,4	0,08	4	*Polygnathus*	100-150 fragmented elements	June 2013, Erlangen
285 JI	18,2	0,06	3	*Polygnathus*	100-150 fragmented elements	January 2020, Erlangen
JI/08	17,6	0,12	3	*Polygnathus*	100-150 fragmented elements	June 2013, Erlangen
1 T	18,1	0,13	3	*Polygnathus*	100-150 fragmented elements	January 2020, Erlangen
2 T	18,3	0,09	3	*Polygnathus*	100-150 fragmented elements	January 2020, Erlangen
3 T	18,3	0,11	3	*Polygnathus*	100-150 fragmented elements	January 2020, Erlangen
4 T	18,0	0,14	3	*Polygnathus*	100-150 fragmented elements	January 2020, Erlangen
5 T	18,1	0,19	3	*Polygnathus*	100-150 fragmented elements	January 2020, Erlangen
UDI/06	18,3	0,08	3	*Polygnathus*	100-150 fragmented elements	June 2013, Erlangen
7 T	18,3	0,10	3	*Polygnathus*	100-150 fragmented elements	January 2020, Erlangen
8 T	18,4	0,11	3	*Polygnathus*	100-150 fragmented elements	January 2020, Erlangen
UDI/09	18,0	0,24	3	*Polygnathus*	100-150 fragmented elements	June 2013, Erlangen
10 T	17,8	0,17	3	*Polygnathus*	100-150 fragmented elements	January 2020, Erlangen
UDI/10	18,1	0,21	3	*Polygnathus*	100-150 fragmented elements	June 2013, Erlangen
UDI/11	18,0	0,26	3	*Polygnathus*	100-150 fragmented elements	June 2013, Erlangen
Wolayer "Glacier" section						
VA 69base	18,0	0,12	3	*Polygnathus*	40 elements	July 2015, Erlangen
69 top	17,7	0,29	3	*Polygnathus*	40 elements	July 2015, Erlangen
70a base	18,0	0,16	3	*Polygnathus*	40 elements	July 2015, Erlangen
5-S (70a top)	18,0	0,02	3	***"Ligonodina"*** ; *Polygnathus*	**2 elements**; 28 elements	June 2013, Erlangen
Zuc di Malaseit Basso (ZMB) section						
ZMB 6 Carlo	18,5	0,09	3	***"Ligonodina"*** ; *Polygnathus*	**1 element**; 24 elements	March 2013, Erlangen
ZMB 34 middle-base	18,7	0,19	3	***"Ligonodina"*** ; *Polygnathus*	**3 elements**; 33 elements	March 2013, Erlangen
ZMB 5 Carlo	19,0		1	*Polygnathus*	11 elements	March 2013, Erlangen
ZMB 26	19,1	0,12	3	*Polygnathus*	40 elements	March 2013, Erlangen
ZMB 4 Carlo	18,8	0,11	2	***"Ligonodina"*** ; *Polygnathus*	**1 element**; 26 elements	March 2013, Erlangen
ZMB 23 top	18,6	0,31	3	*Polygnathus*	44 elements	March 2013, Erlangen
ZMB 20/2	18,2	0,08	2	**coniform**; *Polygnathus*	**1 element**; 40 elements	March 2013, Erlangen
4-S (ZMB 16)	18,6	0,26	3	*Polygnathus*	31 elements	June 2013, Erlangen
ZMB 3 Carlo	18,4	0,10	3	*Polygnathus*	34 elements	March 2013, Erlangen
ZMB 8B	18,4	0,04	2	*Polygnathus*	43 elements	March 2013, Erlangen
ZMB 2 Carlo	18,8	0,14	3	*Polygnathus*	13 elements	March 2013, Erlangen
Val di Collina quarry						
CO/ 1a/ 4	19,5	0,22	3	***"Ligonodina"*** ; ozarkodinid	**4 elements**; 2 elements	March 2013, Erlangen
CO/04/01	19,6	0,18	3	***"Ligonodina"*** ; *Polygnathus*	**6 elements**; 2 elements	March 2013, Erlangen

Supplementary Fig. 2. Excel sheet with published and new δ^{18}O$_{apatite}$ values sorted from N-S according to their paleo-position.

reference	country	area	sample no	stage	biozone	Ma	d^{18}O	correction factors	d^{18}O corrected	paleo-°C	latitude	longitude	paleo-latitude	paleo-longitude	correction d^{18}O$_{sea}$ lat	corrected paleo-°C
Narkiewicz et al., 2017								SIMS corrected -0,6 permil			estimated near Mstislavl town, E of Mogilev					
1	Belarus	Mstislavl 1		Eifelian	*ensensis*		20,60 +/-0,23	-0,6	20,0	22,9	N54°01'14''	E31°43'20''	-10,1	15,73	-0,70	25,3
Narkiewicz et al., 2017								SIMS corrected -0,6 permil			estimated near Kirovsk town, NE of Bobruysk					
2	Belarus	Bobruysk 36		Eifelian	*ensensis*		20,37 +/-0,15	-0,6	19,77	23,94	N53°16'16''	E29°28'32''	-11,62	15,66	-0,67	26,4
Narkiewicz et al., 2017								SIMS corrected -0,6 permil			estimated near Glusha village, W of Bobruysk					
3	Belarus	Glusk 339		Eifelian	*ensensis*		20,38 +/-0,23	-0,6	19,78	23,89	N53°05'15''	E28°51'22''	-12,03	15,61	-0,66	26,4
Narkiewicz et al., 2017								SIMS corrected -0,6 permil			estimated near Zhitkovichi town					
4	Belarus	Zhitkovichi 1		Eifelian	*ensensis*		20,54 +/-0,22	-0,6	19,94	23,17	N52°13'03''	E27°51'19''	-13,02	16,00	-0,64	25,8
Narkiewicz et al., 2017								SIMS corrected -0,6 permil			estimated near Luninets village, E of Pinsk town					
5	Belarus	Pinsk 54-upper		Eifelian	*ensensis*		20,66 +/-0,16	-0,6	20,06	22,63	N52°15'24''	E26°47'48''	-13,53	15,59	-0,63	25,3
6	Belarus	Pinsk 54-lower		Eifelian	*ensensis*		20,77 +/-0,22	-0,6	20,17	22,13						24,9
Joachimski et al., 2009								NBS 120c standard value 22,4 permil			Buggisch and Joachimski, 2006					
7	Germany	Blauer Bruch	BB 08	Givetian	*hemiansatus*	391,68	19,60	-0,7	18,9	27,85	N51°06'47''	E9°08'33''	-22,29	8,63	-0,53	30,6
8	Germany	Blauer Bruch	BB 11	Givetian	*hemiansatus*	391,19	19,50	-0,7	18,8	28,3						31,0
9	Germany	Blauer Bruch	BB 15	Givetian	*hemiansatus*	390,47	18,90	-0,7	18,2	31						33,5
10	Germany	Blauer Bruch	BB 21	Givetian	*hemiansatus*	390,11	19,50	-0,7	18,8	28,3						31,0
11	Germany	Blauer Bruch	BB 22	Givetian	*hemiansatus*	390	19,60	-0,7	18,9	27,85						30,6
12	Germany	Blauer Bruch	BB 24	Givetian	*hemiansatus*	389,71	19,40	-0,7	18,7	28,75						31,4
Königshof et al., 2016								NBS 120c standard value 21,7 permil			Königshof et al., 2016					
13	Germany	Blankenheim, Eifel	BL-12-22	Eifelian	*kockelianus*		19,2	no correction	19,2	26,5	N50°26'29.22''	E6°38'12.79''	-23,79	7,71	-0,54	29,3
14	Germany	Blankenheim, Eifel	BL-12-29c	Eifelian	*kockelianus*		19,2	no correction	19,2	26,5						29,3
Joachimski et al., 2009								NBS 120c standard value 22,4 permil			Weddige, 1977: p. 367 - Bl. Hillesheim, 377 - Bl. Dollendorf					
15	Germany	Eifel, Hillesheimer Mulde	7b/2	Eifelian	*kockelianus*	393,9	19,70	-0,7	19,0	27,4	R 54 650	H 78 110	-23,9	7,83	-0,54	30,1
16	Germany	Eifel, Hillesheimer Mulde	1/3b	Eifelian	*kockelianus*	393,75	20,20	-0,7	19,5	25,15	R 52 960	H 73 640	-23,9	7,83	-0,54	28,0
Joachimski et al., 2009								NBS 120c standard value 22,4 permil			Weddige, 1977: p. 362 - Bl. Schönecken					
17	Germany	Eifel, Schönecken-Dingdorf	P344/D	Eifelian	*kockelianus**	392,59	19,90	-0,7	19,2	26,5	R 32 330 to R 32 320	H 56 620 to H 56 660	-24,1	7,79	-0,54	29,3
18	Germany	Eifel, Schönecken-Dingdorf	P184/D	Givetian	*hemiansatus*	391,25	20,00	-0,7	19,3	26,05					-0,54	28,9
19	Germany	Eifel, Schönecken-Dingdorf	P172/D	Givetian	*hemiansatus*	391,22	19,80	-0,7	19,1	26,95						29,7
20	Germany	Eifel, Schönecken-Dingdorf	P164/D	Givetian	*hemiansatus*	391,2	19,70	-0,7	19,0	27,4						30,1
21	Germany	Eifel, Schönecken-Dingdorf	P162/D	Givetian	*hemiansatus*	391,19	18,50	-0,7	17,8	32,8						35,2
22	Germany	Eifel, Schönecken-Dingdorf	P132/D	Givetian	*hemiansatus*	391,07	19,70	-0,7	19,0	27,4						30,1
23	Germany	Eifel, Schönecken-Dingdorf	P130/D	Givetian	*hemiansatus*	391,06	20,40	-0,7	19,7	24,25						27,2
24	Germany	Eifel, Schönecken-Dingdorf	P90/D	Givetian	*hemiansatus*	390,89	19,30	-0,7	18,6	29,2						31,8
25	Germany	Eifel, Schönecken-Dingdorf	P74/D	Givetian	*hemiansatus*	390,86	19,70	-0,7	19,0	27,4						30,1
26	Germany	Eifel, Schönecken-Dingdorf	P70/D	Givetian	*hemiansatus*	390,86	19,60	-0,7	18,9	27,85						30,6
Elrick et al., 2009								NBS 120c standard value 22,6 permil			Elrick et al., 2009					
27	USA	Antelopes, Nevada		Eifelian	*kockelianus*		18,4	-0,9	17,5	34,15	N39°15'01.1''	W116°14'00.9''	-24,73	-57,34	-0,54	36,4
28	USA	Antelopes, Nevada		Eifelian	*kockelianus*		18,5	-0,9	17,6	33,70						36,0
29	USA	Antelopes, Nevada		Eifelian	*kockelianus*		18,4	-0,9	17,5	34,15						36,4
30	USA	Antelopes, Nevada		Eifelian	*kockelianus*		17,9	-0,9	17	36,40						38,5
31	USA	Antelopes, Nevada		Eifelian	*kockelianus*		18,75	-0,9	17,85	32,58						35,0
32	USA	Antelopes, Nevada		Eifelian	*kockelianus*		19,3	-0,9	18,4	30,1						32,7
33	USA	Antelopes, Nevada		Eifelian	*kockelianus*		19,28	-0,9	18,38	30,19						32,7
34	USA	Antelopes, Nevada		Eifelian	*kockelianus*		18,7	-0,9	17,8	32,8						35,2
35	USA	Antelopes, Nevada		Eifelian	*kockelianus*		18,3	-0,9	17,4	34,6						36,9
this study								NBS 120c standard value 21,7 permil			Vodrážková and Suttner, 2020					
36	Czech Republic	Jirásek quarry section I	30 JI	Eifelian	*kockelianus*		18,2	no correction	18,2	31	N49°54'50.2''	E14°04'34.2''	-27,68	36,03	-0,56	33,4
37	Czech Republic	Jirásek quarry section I	60 M	Eifelian	*kockelianus*		17,9	no correction	17,9	32,35						34,7
38	Czech Republic	Jirásek quarry section I	85 JI	Eifelian	*kockelianus*		18,7	no correction	18,7	28,75						31,3
39	Czech Republic	Jirásek quarry section I	100 M	Eifelian	*kockelianus*		18,1	no correction	18,1	31,45						33,8
40	Czech Republic	Jirásek quarry section I	140 M	Eifelian	*kockelianus*		18,2	no correction	18,2	31						33,4
41	Czech Republic	Jirásek quarry section I	200 M	Eifelian	*kockelianus*		18,2	no correction	18,2	31						33,4
42	Czech Republic	Jirásek quarry section I	Ji/03	Eifelian	*kockelianus*		17,7	no correction	17,7	33,25						35,5

Supplementary Fig. 2. Excel sheet with published and new $\delta^{18}O_{apatite}$ values sorted from N-S according to their paleo-position.

reference	country	area	sample no	stage	biozone	Ma	$d^{18}O$	correction factors	$d^{18}O$ corrected	paleo-°C	latitude	longitude	paleo-latitude	paleo-longitude	correction $d^{18}O_{sea}$ lat	corrected paleo-°C
43	Czech Republic	Jirásek quarry section I	Ji/04	Eifelian	*kockelianus*		17,8	no correction	17,8	32,8						35,1
44	Czech Republic	Jirásek quarry section I	225 JI	Eifelian	*kockelianus*		18,4	no correction	18,4	30,1						32,6
45	Czech Republic	Jirásek quarry section I	240 JI	Eifelian	*kockelianus*		17,8	no correction	17,8	32,8						35,1
46	Czech Republic	Jirásek quarry section I	Ji/05	Eifelian	*kockelianus*		17,8	no correction	17,8	32,8						35,1
47	Czech Republic	Jirásek quarry section I	Ji/06	Eifelian	*kockelianus*		18,1	no correction	18,1	31,45						33,8
48	Czech Republic	Jirásek quarry section I	260 M	Eifelian	*kockelianus*		18,0	no correction	18,0	31,9						34,2
49	Czech Republic	Jirásek quarry section I	Ji/07	Eifelian	*kockelianus*		18,4	no correction	18,4	30,1						32,6
50	Czech Republic	Jirásek quarry section I	285 JI	Eifelian	*kockelianus*		18,2	no correction	18,2	31						33,4
51	Czech Republic	Jirásek quarry section I	Ji/08	Eifelian	*kockelianus*		17,6	no correction	17,6	33,7						35,9
52	Czech Republic	Jirásek quarry section I	1 T	Eifelian	*ensensis*		18,1	no correction	18,1	31,45						33,8
53	Czech Republic	Jirásek quarry section I	2 T	Eifelian	*ensensis*		18,3	no correction	18,3	30,55						33,0
54	Czech Republic	Jirásek quarry section I	3 T	Eifelian	*ensensis*		18,3	no correction	18,3	30,55						33,0
55	Czech Republic	Jirásek quarry section I	4 T	Eifelian	*ensensis*		18,0	no correction	18,0	31,9						34,2
56	Czech Republic	Jirásek quarry section I	5 T	Eifelian	*ensensis*		18,1	no correction	18,1	31,45						33,8
57	Czech Republic	Jirásek quarry section I	UDI/06	Eifelian	*ensensis*		18,3	no correction	18,3	30,55						33,0
58	Czech Republic	Jirásek quarry section I	7 T	Eifelian	*ensensis*		18,3	no correction	18,3	30,55						33,0
59	Czech Republic	Jirásek quarry section I	8 T	Eifelian	*ensensis*		18,4	no correction	18,4	30,1						32,6
60	Czech Republic	Jirásek quarry section I	UDI/09	Eifelian	*ensensis*		18,0	no correction	18,0	31,9						34,2
61	Czech Republic	Jirásek quarry section I	10 T	Eifelian	*ensensis*		17,8	no correction	17,8	32,8						35,1
62	Czech Republic	Jirásek quarry section I	UDI/10	Eifelian	*ensensis*		18,1	no correction	18,1	31,45						33,8
63	Czech Republic	Jirásek quarry section I	UDI/11	Eifelian	*ensensis*		18,0	no correction	18,0	31,9						34,2
this study								NBS 120c standard value 21,7 permil			this study					
64	Austria	Wolayer "Glacier" section	VA 69base	Eifelian	*ensensis*		18,0	no correction	18,0	31,9	N46°36'46.56''	E12°52'33.66''	-34,38	39,47	-0,66	33,8
65	Austria	Wolayer "Glacier" section	69 top	Eifelian	*ensensis*		17,7	no correction	17,7	33,25						35,1
66	Austria	Wolayer "Glacier" section	70a base	Givetian	*hemiansatus*		18,0	no correction	18,0	31,9						33,8
67	Austria	Wolayer "Glacier" section	5-S (70a top)	Givetian	*hemiansatus*		18,0	no correction	18,0	31,9						33,8
this study								NBS 120c standard value 21,7 permil			this study					
68	Italy	ZMB section	ZMB 6 Carlo	Eifelian	*kockelianus*		18,5	no correction	18,5	29,65	N46°33'19.06''	E13°11'10.6''	-34,47	39,71	-0,66	31,7
69	Italy	ZMB section	ZMB 34 m b	Eifelian	*kockelianus*		18,7	no correction	18,7	28,75						30,9
70	Italy	ZMB section	ZMB 5 Carlo	Eifelian	*kockelianus*		19,0	no correction	19,0	27,4						29,6
71	Italy	ZMB section	ZMB 26	Eifelian	*kockelianus*		19,1	no correction	19,1	26,95						29,2
72	Italy	ZMB section	ZMB 4 Carlo	Eifelian	*kockelianus*		18,8	no correction	18,8	28,3						30,5
73	Italy	ZMB section	ZMB 23 top	Eifelian	*kockelianus*		18,6	no correction	18,6	29,2						31,3
74	Italy	ZMB section	ZMB 20/2	Eifelian	*kockelianus*		18,2	no correction	18,2	31						33,0
75	Italy	ZMB section	4-S (ZMB 16)	Eifelian	*ensensis*		18,6	no correction	18,6	29,2						31,3
76	Italy	ZMB section	ZMB 3 Carlo	Eifelian	*ensensis*		18,4	no correction	18,4	30,1						32,1
77	Italy	ZMB section	ZMB 8B	Givetian	*hemiansatus*		18,4	no correction	18,4	30,1						32,1
78	Italy	ZMB section	ZMB 2 Carlo	Givetian	*timorensis*		18,8	no correction	18,8	28,3						30,5
this study								NBS 120c standard value 21,7 permil			this study					
79	Italy	Val di Collina quarry	CO/ 1a/ 4	Eifelian	*ensensis*		19,5	no correction	19,5	25,15	N46°35'49.07''	E12°55'29.22''	-34,4	39,51	-0,66	27,5
80	Italy	Val di Collina quarry	CO/04/01	Eifelian	*ensensis*		19,6	no correction	19,6	24,7						27,1
Joachimski et al., 2009								NBS 120c standard value 22,4 permil			Buggisch and Joachimski, 2006					
81	France	Pic de Bissous	MN 99	Eifelian	*kockelianus*	393,94	20,40	-0,7	19,7	24,25	N43°36'10.0''	E3°21'28.9''	-36,35	30,98	-0,70	26,5
82	France	Pic de Bissous	MN 100	Eifelian	*kockelianus*	393,69	20,60	-0,7	19,9	23,35						25,7
83	France	Pic de Bissous	MN 101	Eifelian	*kockelianus*	393,34	19,70	-0,7	19,0	27,4						29,5
84	France	Pic de Bissous	MN 102	Eifelian	*kockelianus*	393,1	20,90	-0,7	20,2	22						24,4
85	France	Pic de Bissous	MN 103	Eifelian	*kockelianus**	392,52	20,70	-0,7	20,0	22,9						25,3
86	France	Pic de Bissous	MN 104	Eifelian	*kockelianus**	392,22	20,30	-0,7	19,6	24,7						26,9
87	France	Pic de Bissous	MN 105	Givetian	*hemiansatus*	391,7	20,70	-0,7	20,0	22,9						25,3
88	France	Pic de Bissous	MN 106	Givetian	*hemiansatus*	390,54	20,90	-0,7	20,2	22						24,4
89	France	Pic de Bissous	MN 107	Givetian	*hemiansatus*	390,09	21,10	-0,7	20,4	21,1						23,6

Remark: *kockelianus* * resembles the age published by Joachimski et al. (2009).
It has been corrected to *ensensis* Biozone according to Weddige (1977: p. 373, Tab. 11) for the Schönecken-Dingdorf section (Eifel) and the absolute age mentioned in the stratigraphic chart of Gradstein et al. (2004, 2008) for Pic de Bissous (France).

Supplementary Fig. 3. Conodont Colour Alteration Index (CAI) representative assemblages of each section (lower right: sample number).

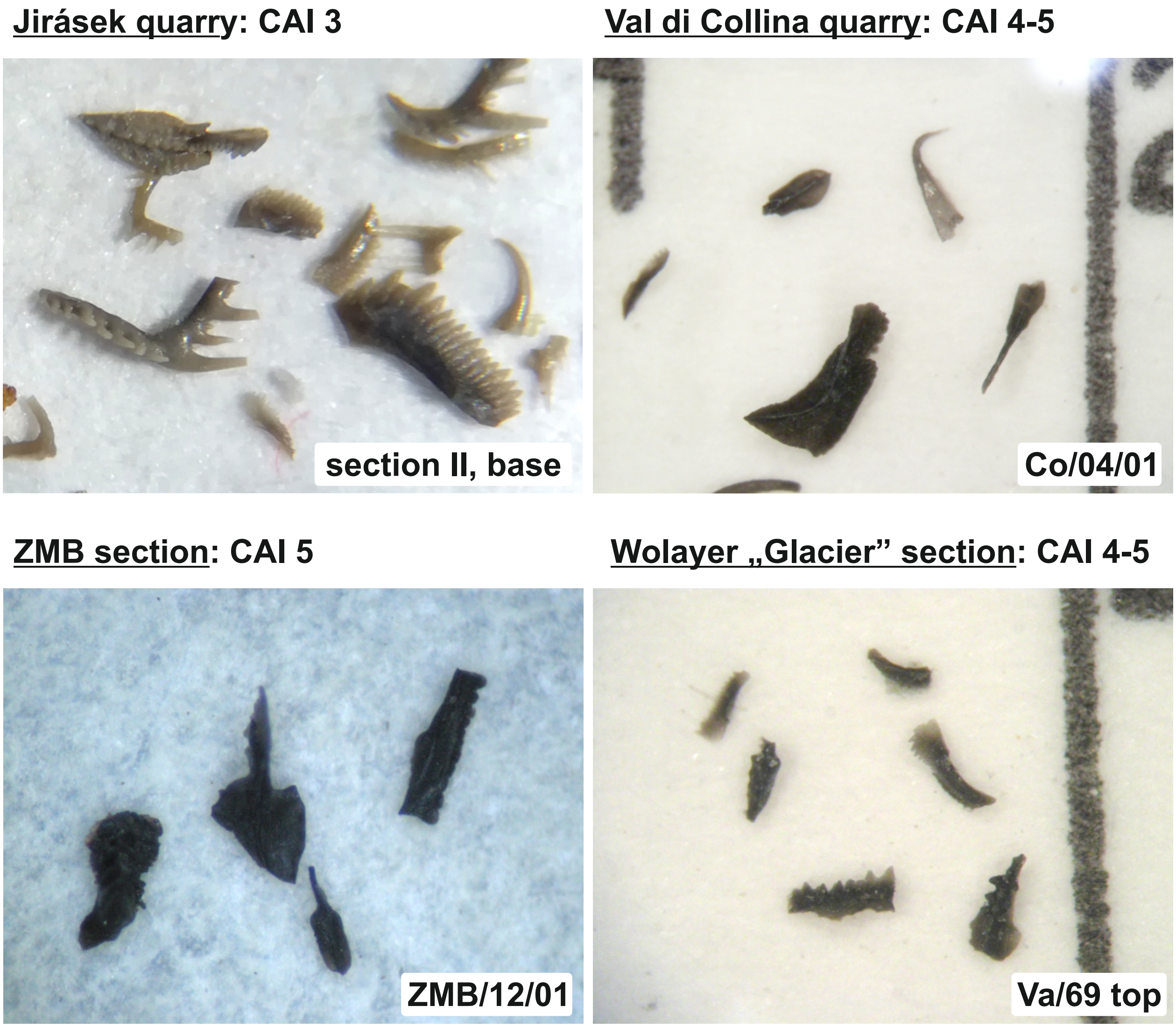

Supplement: Supplementary file 1 — Supplementary Figures. [file 41598_2021_96013_MOESM1_ESM.pdf]
